# Multi-Year Molecular Survey of *Trypanosoma* spp. in Wild Micromammals at 18 Surveillance Sites in the Republic of Korea: First Molecular Evidence of a *Trypanosoma lewisi*-like Genotype

**DOI:** 10.3390/ani16182832

**Published:** 2026-09-09

**Authors:** Insu Choi, Beoul Kim, You-Jeong Lee, Hyo-Min Woo, Jae-Woo Choi, Man Hee Rhee, Yong-Myung Kang, Dongmi Kwak, Min-Goo Seo

**Affiliations:** College of Veterinary Medicine and Institute for Veterinary Biomedical Science, Kyungpook National University, Daegu 41566, Republic of Korea; cis200232@naver.com (I.C.); kbjjhnm@naver.com (B.K.); wowgirlsgood@naver.com (Y.-J.L.); whmsm9902@naver.com (H.-M.W.); choiwoo777@naver.com (J.-W.C.); rheemh@knu.ac.kr (M.H.R.); kamaboy88@knu.ac.kr (Y.-M.K.); dmkwak@knu.ac.kr (D.K.)

**Keywords:** *Trypanosoma grosi*, *Trypanosoma lewisi*-like genotype, *Apodemus agrarius*, molecular epidemiology, wild micromammals, Republic of Korea

## Abstract

Rodent-associated *Trypanosoma* spp. are widely distributed in wild rodents, but their epidemiology in the Republic of Korea remains incompletely characterized. This study investigated the prevalence, spatiotemporal distribution, and genetic characteristics of *Trypanosoma* spp. in 1169 wild micromammals representing 12 taxa, including 1010 *Apodemus agrarius*, collected from 18 sites across the Republic of Korea between 2022 and 2025 using molecular methods. *Trypanosoma* spp. DNA was detected in 58 (5.7%) *Apodemus agrarius*; all other micromammal species examined were negative. Fifty-seven samples were identified as *T. grosi* and one sequence was designated as a *T. lewisi*-like genotype. No significant regional differences in prevalence were observed. Annual prevalence estimates were presented descriptively because the seasonal and geographic composition of sampling differed among study years. When the seasonal comparison was restricted to 2022, 2023, and 2025, prevalence was significantly higher in autumn than in spring. The single *T. lewisi*-like genotype showed 98.5–99.7% nucleotide identity with reference *T. lewisi* sequences and clustered within the *T. lewisi* clade. Because species assignment was based on a single genetic marker, the sequence was conservatively designated as a *T. lewisi*-like genotype. To the best of our knowledge, this study provides the first molecular evidence of a *T. lewisi*-like genotype in a wild micromammal in the Republic of Korea. These findings provide updated epidemiological information on rodent-associated *Trypanosoma* spp. in the Republic of Korea.

## Simple Summary

Wild micromammals, such as mice and shrews, can harbor *Trypanosoma* parasites, some of which have potential relevance to animal and human health. However, nationwide information on rodent-associated *Trypanosoma* species in the Republic of Korea remains limited. In this study, molecular methods were used to examine 1169 wild micromammals collected from 18 sites between 2022 and 2025. *Trypanosoma* spp. were detected in 58 animals, all of which were *Apodemus agrarius* (striped field mice). *Trypanosoma grosi* was the predominant species, whereas a single *T. lewisi*-like genotype was detected, representing the first molecular evidence of such a genotype in a wild micromammal in the Republic of Korea. Observed prevalence varied among years, and prevalence was higher in autumn than in spring; however, these differences should be interpreted cautiously because the sampling design was not fully uniform across the study period. These findings provide baseline information for continued wildlife surveillance and future studies of rodent-associated trypanosomes.

## 1. Introduction

Wild micromammals, including rodents and insectivores, can serve as hosts for diverse zoonotic pathogens and contribute to the maintenance of infectious agents in natural ecosystems [1,2,3,4]. Rodents (order Rodentia) represent the largest and most diverse mammalian order and are widely distributed across all continents except Antarctica [5]. Insectivorous mammals of the order Eulipotyphla can also harbor diverse microorganisms; however, their roles in pathogen ecology remain less well characterized than those of rodents [6].

The genus *Trypanosoma* (family Trypanosomatidae, class Kinetoplastea) comprises flagellated protozoan parasites infecting a wide range of vertebrate hosts, including humans, domestic animals, and wildlife [7]. Based on their developmental site within invertebrate vectors, trypanosomes have traditionally been divided into Salivaria and Stercoraria [8]. Salivarian trypanosomes develop primarily in the anterior part of the vector and are transmitted through vector feeding, whereas stercorarian trypanosomes develop in the posterior part of the vector and are typically transmitted through contamination with vector excreta [8]. Among stercorarian trypanosomes, *T. cruzi* is the major species associated with severe human disease. Rodent-associated species of the subgenus *Herpetosoma*, including *T. grosi* and *T. lewisi*, have generally been considered to have limited pathogenicity; however, sporadic human infections with *T. lewisi* or closely related trypanosomes have been reported.

Among rodent-associated trypanosomes, *T. grosi* is frequently associated with *Apodemus* spp. and has been reported in wild micromammals from several European and Asian countries, including the United Kingdom [9], Ireland [10], Germany [11], Hungary [12], the Czech Republic [13], China [14], and Japan [15]. In contrast, *T. lewisi* is classically associated with *Rattus* spp. and is widely distributed in rodent populations worldwide. Although human infection with *T. lewisi* is uncommon, sporadic cases have been reported, supporting its potential public health relevance [16].

In the Republic of Korea (ROK), molecular studies of *Trypanosoma* infection in wild micromammals remain limited. A previous nationwide survey based on samples collected in 2021 identified *T. grosi* in wild micromammals and provided the first molecular evidence of rodent-associated trypanosomes in the country [17]. However, that investigation was based on samples collected during a single year, limiting assessment of temporal variation. To our knowledge, molecular evidence of a *T. lewisi*-like genotype in wild micromammals has not previously been reported in the ROK. Consequently, multi-year patterns in the prevalence, geographic distribution, and species diversity of rodent-associated *Trypanosoma* spp. in the ROK remain insufficiently characterized.

To address these knowledge gaps, this study investigated the molecular prevalence and spatiotemporal distribution of *Trypanosoma* spp. in 1169 wild micromammals collected from 18 sites across the ROK between 2022 and 2025. PCR-positive samples were further characterized by sequencing and phylogenetic analysis to determine their genetic identity and assess the genetic relationships of the detected trypanosomes with previously reported sequences.

## 2. Materials and Methods

### 2.1. Ethical Approval

Micromammals used in this study were collected as part of a nationwide surveillance program conducted by the Korea Disease Control and Prevention Agency (KDCA). All animal handling and sampling procedures were conducted in accordance with institutional guidelines for animal care and use and were approved by the Institutional Animal Care and Use Committee (IACUC) of the KDCA (approval no. KDCA-093-18). The use and analysis of the collected samples in the present study were additionally approved by the IACUC of Kyungpook National University (approval no. KNU-2022-0441).

### 2.2. Micromammal Collection

Micromammals were collected from 18 sites across the ROK through a nationwide surveillance program conducted in collaboration with the KDCA. Sampling was conducted during spring and autumn in 2022, 2023, and 2025 and during autumn in 2024. A total of 11 sampling rounds were conducted: two rounds each in spring and autumn of 2022 and 2023, one round in autumn 2024, and one round each in spring and autumn 2025. Sampling at each site encompassed five habitat types: paddy fields, farmland, waterways, forests, and reservoirs. During each sampling round, 100 Sherman live traps (3 × 3 × 9 inches; BioQuip, Compton, CA, USA) were deployed at each participating site, with 20 traps placed in each habitat type at intervals of 3–5 m. The traps were baited with peanut butter–coated cookies, set before sunset, and retrieved the following morning.

Captured micromammals were identified to the lowest feasible taxonomic level using standard taxonomic keys [18]. Animals were euthanized by CO_2_ inhalation in accordance with institutional animal welfare guidelines, after which blood samples were collected by cardiac puncture. Although rodents (order Rodentia) comprised the majority of captures, four taxa of the order Eulipotyphla—*Crocidura lasiura*, *C. suaveolens*, *Crocidura* spp., and *Mogera robusta*—were also collected and included in the study.

**Figure 1:**
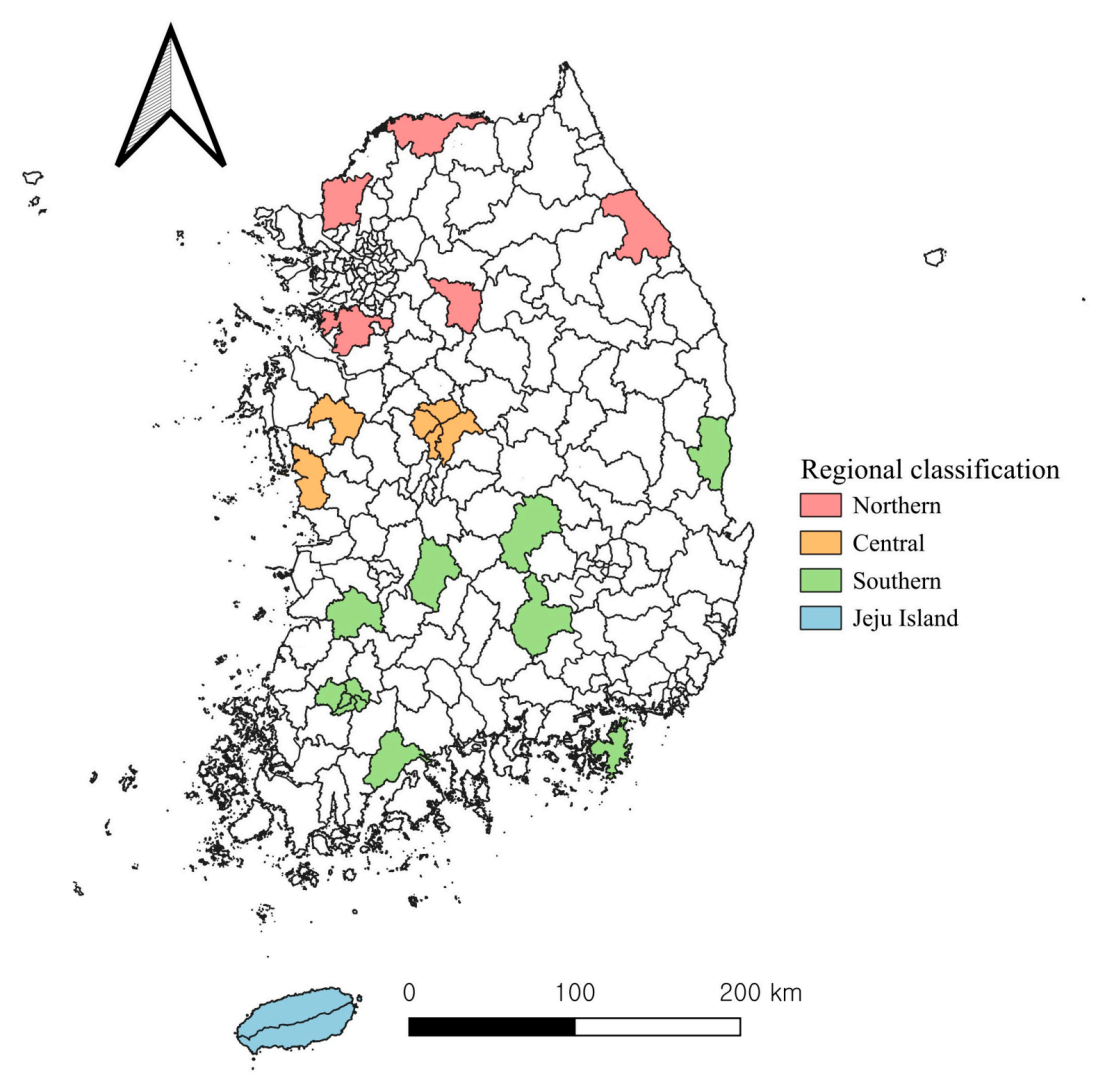
Geographic distribution and regional classification of the sampling sites in the Republic of Korea. Sampling sites were classified into four regions: Northern (red), Central (orange), Southern (green), and Jeju Island (blue).

Sampling sites were grouped into four geographic regions: Northern (Cheorwon, Gangneung, Yeoju, Hwaseong, and Paju), Central (Cheongju, Boryeong, and Yesan), Southern (Jeongeup, Boseong, Jinan, Geoje, Hapcheon, Gimcheon, Yeongdeok, and Gwangju), and Jeju Island (Seogwipo and Jeju). Two additional sampling sites, Gwangju and Jeju, were included beginning in 2025. The geographic distribution of the sampling sites is shown in Figure 1.

### 2.3. DNA Extraction and Molecular Detection

DNA extraction, PCR amplification, and post-PCR analysis were performed in separate laboratory areas to minimize the risk of contamination. Genomic DNA was extracted from whole blood samples using the Clear-S™ Quick DNA Kit (InVirusTech, Gwangju, Republic of Korea) according to the manufacturer’s instructions. *Trypanosoma* spp. DNA was detected by PCR amplification of the internal transcribed spacer 1 (ITS1) region using TRYP1R (5′-GGAAGCCAAGTCATCCATCG-3′) and TRYP1S (5′-CGTCCCTGCCATTTGTACACAC-3′) primers to amplify an approximately 623-bp fragment [19]. PCR was performed in a total reaction volume of 20 μL containing 1 μL of each primer (10 pmol/μL), 3 μL of genomic DNA, and 15 μL of distilled water using an AccuPower HotStart PCR PreMix (Bioneer, Daejeon, Republic of Korea). The amplification conditions consisted of an initial denaturation at 95 °C for 5 min, followed by 35 cycles of denaturation at 95 °C for 30 s, annealing at 55 °C for 1 min, and extension at 72 °C for 1 min, with a final extension at 72 °C for 10 min. PCR products were analyzed by agarose gel electrophoresis to confirm amplification of the expected fragment. Positive and negative controls were included in each PCR run.

### 2.4. DNA Sequencing and Phylogenetic Analysis

PCR-positive products were purified and bidirectionally sequenced by Macrogen (Seoul, Republic of Korea). The resulting sequences were compared with reference sequences available in the NCBI GenBank database using BLAST web service (National Center for Biotechnology Information, Bethesda, MD, USA; accessed on 1 March 2026). Species assignment was based on nucleotide sequence similarity and phylogenetic placement relative to reference sequences. Multiple sequence alignment of the study and reference sequences was performed using CLUSTAL Omega version 1.2.1. Sequence alignments were inspected using BioEdit version 7.2.5, and identical redundant sequences were excluded from the phylogenetic analysis. Representative sequences were selected to reflect different sampling years and geographic regions. Phylogenetic analysis was performed using MEGA version 7.0 [20]. An unrooted phylogenetic tree was reconstructed using the maximum likelihood method under the Kimura two-parameter model, and branch support was evaluated using 1000 bootstrap replicates.

### 2.5. Statistical Analysis

Statistical analyses were performed using GraphPad Prism version 5.04 (GraphPad Software Inc., La Jolla, CA, USA). Analyses of *Trypanosoma* spp. prevalence were restricted to *Apodemus agrarius*. Regional differences were assessed using Pearson’s chi-square test. Annual prevalence was presented descriptively. Seasonal differences were assessed using Fisher’s exact test using data from 2022, 2023, and 2025 only. Exact binomial (Clopper–Pearson) 95% confidence intervals (CIs) were calculated, and *p* < 0.05 was considered statistically significant.

### 2.6. Geographical Analysis

Geographical mapping and visualization were performed using QGIS version 3.40 (QGIS Development Team) [21]. Maps were generated to display the geographic distribution and regional classification of sampling sites and the number of *Trypanosoma* spp.-positive *A. agrarius* detected at each site.

## 3. Results

### 3.1. Taxonomic Composition of Micromammals

A total of 1169 micromammals representing 12 taxa were collected from 18 sampling sites during the study period (Table 1). Rodents (order Rodentia) accounted for the majority of captures, with *Apodemus agrarius* being the predominant taxon, accounting for 86.4% (1010/1169) of all examined micromammals. The remaining rodent taxa, including *Apodemus peninsulae*, *Craseomys regulus*, *Craseomys rufocanus*, *Micromys minutus*, *Microtus fortis*, *Myodes regulus*, and *Tscherskia triton*, were represented at substantially lower frequencies. Eulipotyphla comprised *Crocidura lasiura*, *Crocidura suaveolens*, *Crocidura* spp., and *Mogera robusta*. The distribution of captured micromammals by habitat was 23.0% in waterways, 22.2% in forests, 21.7% in reservoirs, 18.9% in farmland, and 14.2% in paddy fields.

### 3.2. Prevalence of Trypanosoma spp.

*Trypanosoma* spp. DNA was detected in 58 of 1010 *A. agrarius* examined, corresponding to a prevalence of 5.7% (95% CI: 4.4–7.4%). Among these, 57 were positive for *T. grosi* (5.6%; 95% CI: 4.3–7.3%), whereas the remaining sample yielded a sequence conservatively designated as a *T. lewisi*-like genotype (0.1%; 95% CI: 0.003–0.55%). No *Trypanosoma* spp. DNA was detected in the other micromammal taxa examined (Table 1).

**Figure 2:**
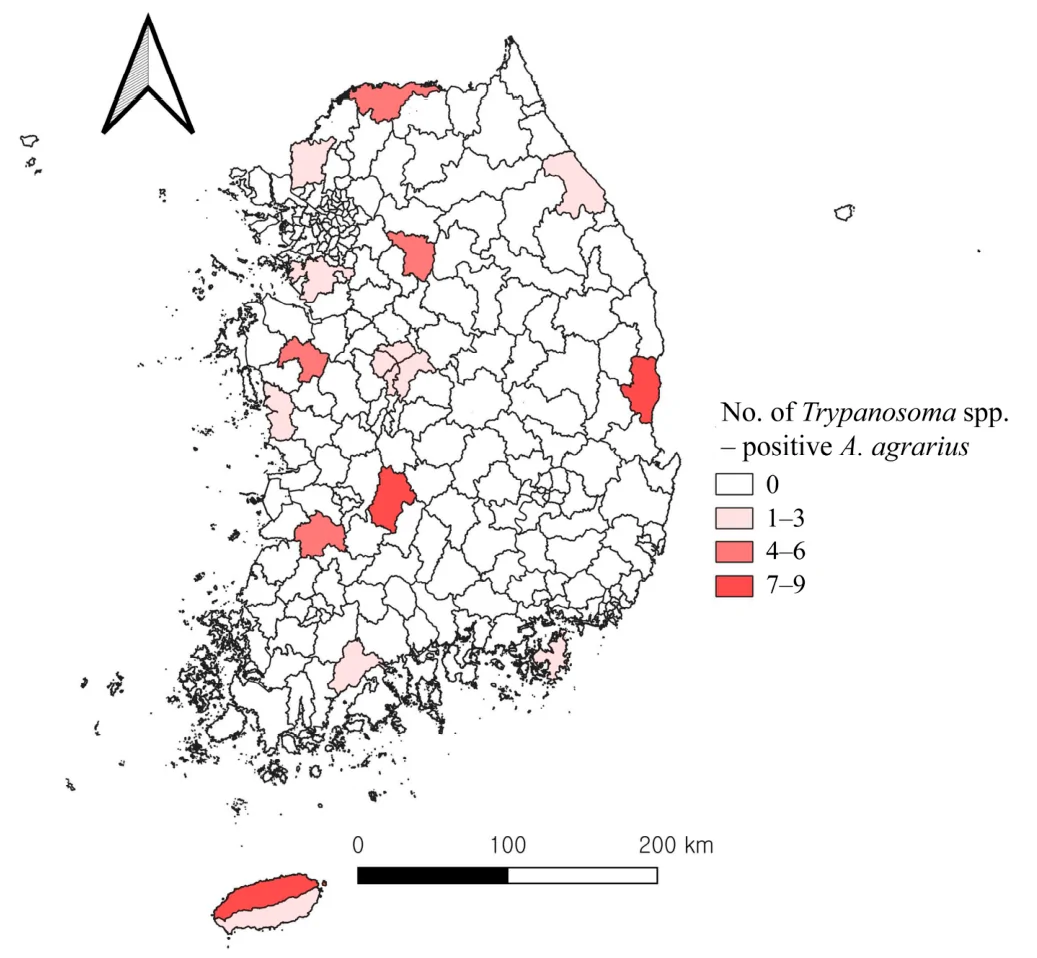
Geographic distribution of *Trypanosoma* spp.-positive *Apodemus agrarius* in the Republic of Korea. Shading indicates the number of *Trypanosoma* spp.-positive *A. agrarius* detected at each sampling site.

Regional prevalence in *A. agrarius* was highest on Jeju Island (8.1%; 12/149; 95% CI: 4.2–13.6%), followed by the Central (6.6%; 8/121; 95% CI: 2.9–12.6%), Southern (5.8%; 22/378; 95% CI: 3.7–8.7%), and Northern regions (4.4%; 16/362; 95% CI: 2.5–7.1%). However, the overall difference among regions was not statistically significant (χ^2^ = 2.813, df = 3, *p* = 0.421) (Table 2; Figure 2).

**Figure 3:**
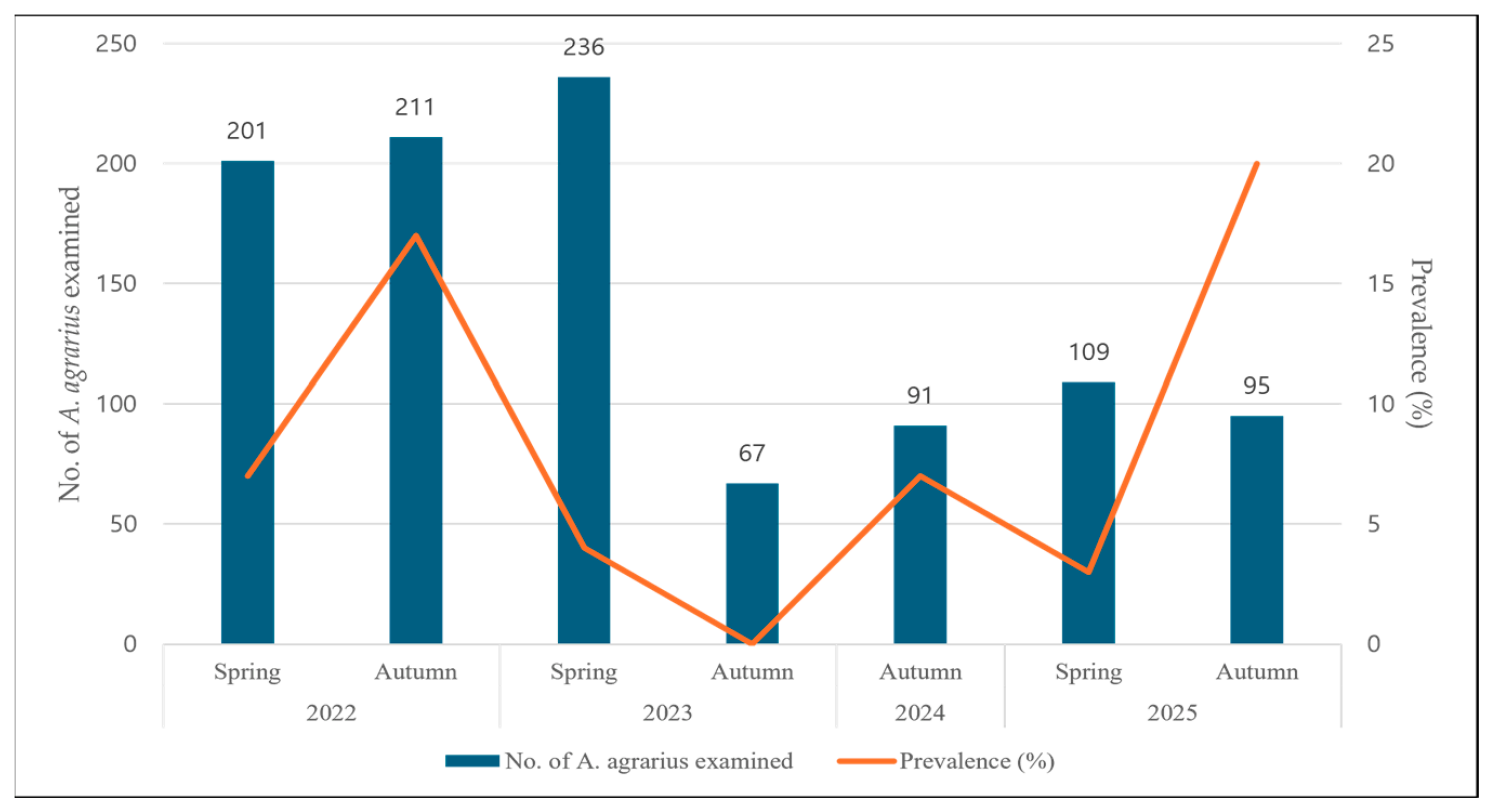
Number of *Apodemus agrarius* examined and prevalence of *Trypanosoma* spp. by year and season in the Republic of Korea. Bars represent the number of *A. agrarius* examined, and the line represents *Trypanosoma* spp. prevalence (%). Values for spring and autumn 2022 and 2023 represent data aggregated from two sampling rounds per season; sampling in 2024 was conducted only in autumn, whereas one sampling round was conducted in each season in 2025. The 2024 data were excluded from the statistical comparison between spring and autumn.

The observed annual prevalence in *A. agrarius* was 5.8% (24/412; 95% CI: 3.8–8.5%) in 2022, 1.3% (4/303; 95% CI: 0.4–3.3%) in 2023, 7.7% (7/91; 95% CI: 3.1–15.2%) in 2024, and 11.3% (23/204; 95% CI: 7.3–16.4%) in 2025 (Table 2; Figure 3). Because the seasonal and geographic composition of sampling differed among study years, these annual prevalence estimates were considered descriptive and were not interpreted as evidence of an independent year effect.

For the seasonal comparison, only data from 2022, 2023, and 2025, when sampling was conducted during both spring and autumn, were included. The prevalence of *Trypanosoma* spp. in *A. agrarius* was 2.6% (14/546; 95% CI: 1.4–4.3%) in spring and 9.9% (37/373; 95% CI: 7.1–13.4%) in autumn. Prevalence was significantly higher in autumn than in spring (Fisher’s exact test, *p* < 0.001) (Table 2; Figure 3).

### 3.3. Genetic and Phylogenetic Analyses

**Figure 4:**
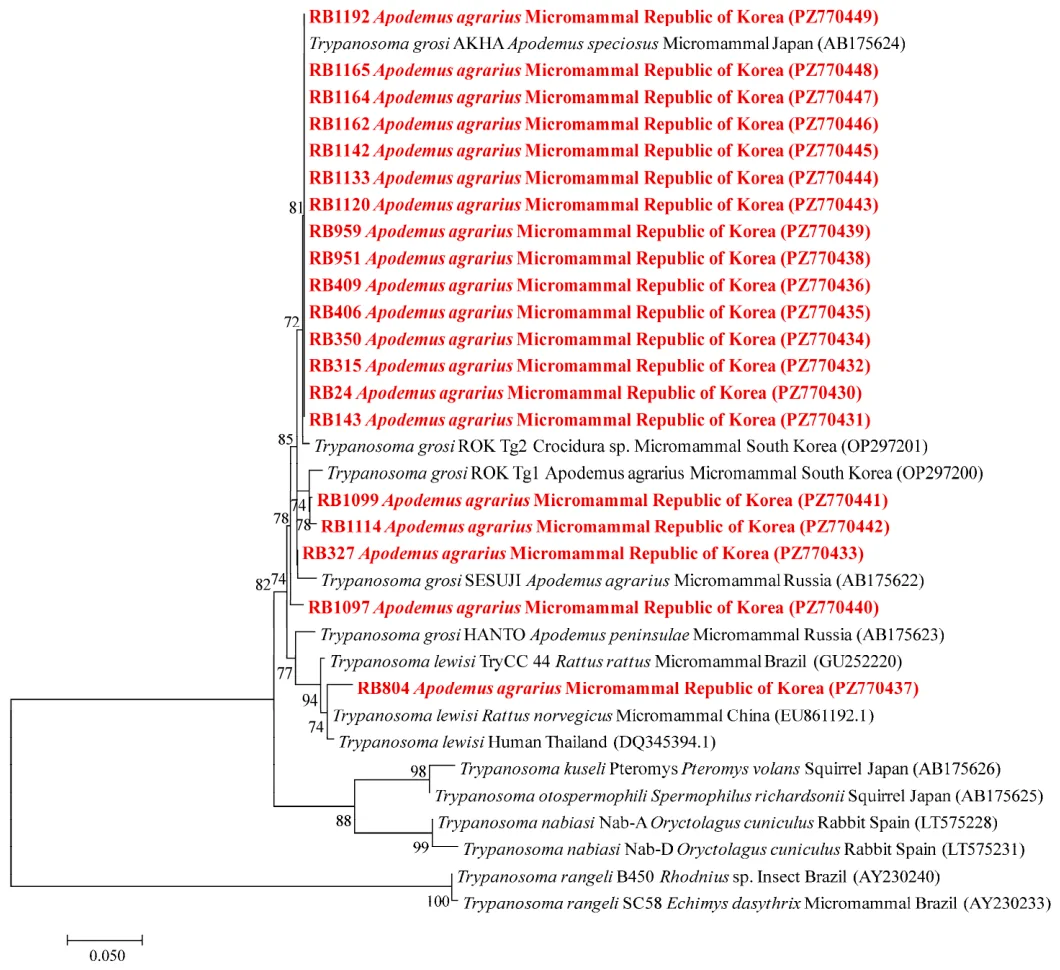
Phylogenetic relationships of *Trypanosoma* spp. based on ITS1 sequences. Sequences obtained in the present study are highlighted in red and labeled with their corresponding GenBank accession numbers. An unrooted phylogenetic tree was reconstructed using the maximum likelihood method under the Kimura two-parameter model with 1000 bootstrap replicates. Bootstrap values are shown at the nodes. The scale bar indicates the number of nucleotide substitutions per site.

Twenty representative sequences were selected from the 58 PCR-positive samples for phylogenetic analysis to represent different sampling years and geographic regions while excluding redundant identical sequences. Based on nucleotide sequence similarity and phylogenetic placement, 19 representative sequences were assigned to *T. grosi*, whereas one sequence was conservatively designated as a *T. lewisi*-like genotype. The 19 representative *T. grosi* sequences showed 96.7–100% nucleotide identity with one another and 95.5–100% identity with reference *T. grosi* sequences available in GenBank. The single *T. lewisi*-like genotype showed 98.5–99.7% nucleotide identity with reference *T. lewisi* sequences available in GenBank and clustered within the *T. lewisi* clade in the phylogenetic analysis (Figure 4). The 20 representative sequences generated in this study were deposited in GenBank under accession numbers PZ770430–PZ770449.

## 4. Discussion

This multi-year molecular survey provides updated epidemiological data on rodent-associated *Trypanosoma* spp. in wild micromammals from the ROK. Compared with the previous nationwide survey [17], the present study included a larger sample size and samples collected from 2022 to 2025. Sampling was conducted during both spring and autumn in 2022, 2023, and 2025, whereas only autumn sampling was performed in 2024. *T. grosi* was the predominant species detected, whereas a single *T. lewisi*-like genotype was identified in *A. agrarius*.

The predominance of *T. grosi* observed in this study is consistent with previous reports documenting this species in *Apodemus* spp. across Europe and Asia [22]. In East Asia, *T. grosi* has been reported in *A. agrarius* in China [14] and the ROK [17], as well as in *Apodemus speciosus* in Japan [23]. These regional findings are consistent with the detection of *T. grosi* predominantly in *A. agrarius* in the present study. *T. lewisi*, in contrast, is most commonly associated with *Rattus* spp., with fleas serving as its principal vectors [24]. Although human infection is uncommon, sporadic infections with *T. lewisi* or *T. lewisi*-like trypanosomes have been reported, including cases in infants [25,26,27]. However, because only a single *T. lewisi*-like genotype was detected in the present study, its host association and epidemiological significance should be interpreted cautiously.

All *Trypanosoma*-positive micromammals identified in the present study were *A. agrarius*. *T. grosi* has previously been detected in *A. agrarius* in the ROK, with a prevalence of 26.5% (77/290) in a nationwide survey conducted in 2021 [17]. In the present study, the prevalence of *T. grosi* in *A. agrarius* was lower (5.6%, 57/1010). However, this difference should not be interpreted as evidence of a temporal decline because the studies differed in sampling year, geographic and seasonal sampling composition, and laboratory procedures. Potential contributors to the difference include interannual variation in host or vector populations, ecological conditions, sampling structure, and methodological differences between studies; however, these factors were not directly evaluated. *A. agrarius* was also the predominant micromammal in the present survey, accounting for 86.4% of the animals examined, which should be considered when interpreting the apparent host distribution of *Trypanosoma* spp.

A previous nationwide study in the ROK also detected *T. grosi* in *Crocidura* sp. (8.7%, 2/23) [17], whereas no *Trypanosoma*-positive Eulipotyphla were identified in the present study. The absence of detection in these taxa does not necessarily indicate a lack of host involvement, particularly because several non-*A. agrarius* taxa were represented by relatively small sample sizes. In Southeast Asia, rodent-associated *Trypanosoma* spp. have been molecularly detected in *Rattus norvegicus* in Malaysia [28], *Bandicota indica* in Thailand [29], and *R. norvegicus* and *Rattus tanezumi* in Indonesia [30]. In contrast, the single *T. lewisi*-like genotype detected in the present study was identified in *A. agrarius*. A single detection is insufficient to determine whether *A. agrarius* represents a competent reservoir host, an incidental host, or a sporadically infected host, and additional surveillance is required to clarify the epidemiological significance of this finding.

Among *A. agrarius*, prevalence was numerically highest on Jeju Island, although the overall difference among geographic regions was not statistically significant. Interestingly, a previous nationwide survey also reported a high prevalence of *T. grosi* in wild micromammals collected from Seogwipo, Jeju Island (48.4%, 15/31) [17]. However, direct comparison between the two studies should be made cautiously because of differences in sampling periods and study design. Fleas are important vectors of rodent-associated trypanosomes [24], and environmental factors such as temperature, humidity, and season can influence flea abundance and population dynamics [31]. These factors may contribute to geographic variation in *Trypanosoma* transmission; however, environmental variables and flea populations were not assessed in the present study. Therefore, the higher observed prevalence on Jeju Island should be interpreted cautiously and should not be considered evidence of an independent regional effect.

Annual prevalence in *A. agrarius* was 5.8% in 2022, 1.3% in 2023, 7.7% in 2024, and 11.3% in 2025. However, these values were interpreted descriptively because the seasonal and geographic composition of sampling differed among years. Sampling effort was not uniform across years: two sampling rounds were conducted in each season in 2022 and 2023, only autumn was sampled in 2024, and one sampling round was conducted in each season in 2025. Therefore, the observed annual variation should not be interpreted as evidence of an independent year effect. Further studies using a standardized longitudinal sampling design are needed to clarify interannual variation in *Trypanosoma* prevalence.

When the seasonal comparison was restricted to *A. agrarius* collected in 2022, 2023, and 2025, prevalence was significantly higher in autumn (9.9%) than in spring (2.6%). Fleas are important vectors of several rodent-associated trypanosomes [24,32], suggesting that seasonal variation in vector activity may contribute to temporal patterns of infection. In a longitudinal study of *Trypanosoma* (*Herpetosoma*) *microti* in field voles, trypanosome prevalence was positively associated with flea prevalence recorded three months earlier, with the highest prevalence occurring in autumn [32]. This finding provides a plausible ecological explanation for the higher prevalence observed during autumn in the present study. However, flea abundance and other vector-related factors were not measured. Therefore, the observed seasonal difference should not be interpreted as evidence of an independent seasonal effect. Further longitudinal studies incorporating standardized seasonal sampling and simultaneous flea surveillance are needed.

Phylogenetic analysis showed that the *T. grosi* sequences identified in this study clustered with previously reported reference *T. grosi* sequences from the ROK [17], Japan and Russia [15], supporting their molecular assignment to this species. The single *T. lewisi*-like genotype showed 98.5–99.7% nucleotide identity with reference *T. lewisi* sequences and clustered within the *T. lewisi* clade. Because identification was based on a single genetic marker (ITS1), the sequence was conservatively designated as a *T. lewisi*-like genotype. It was also phylogenetically related to a *T. lewisi*-like sequence detected in a human infant in Thailand [25]. However, phylogenetic relatedness to sequences detected in humans does not, by itself, demonstrate zoonotic transmission or indicate an immediate public health risk. Therefore, this finding should be regarded as baseline molecular evidence of a *T. lewisi*-like genotype in a wild micromammal in the ROK. More broadly, the present study provides baseline molecular information on the occurrence of *Trypanosoma* DNA in blood samples from wild micromammals.

This study has several limitations. First, detection of *Trypanosoma* infection was based on molecular methods, and microscopic examination was not performed to assess parasitemia or parasite morphology. Although PCR-based methods can provide greater sensitivity than microscopy, particularly when parasitemia is low [33], combining molecular and morphological approaches would provide more comprehensive parasite characterization. Second, only blood samples were analyzed, and ectoparasites, such as fleas, were not collected or examined. Because fleas are important vectors of several rodent-associated trypanosomes [24], simultaneous investigation of mammalian hosts and their ectoparasites would provide a more comprehensive understanding of transmission dynamics. Third, the sampling design was not fully balanced across years and seasons, and two additional sampling sites were included in 2025. Consequently, the observed annual and seasonal differences may partly reflect variation in sampling effort and site composition and should not be interpreted as independent effects of year or season. Fourth, eight non-*A. agrarius* taxa were represented by only 1–5 individuals; therefore, the absence of *Trypanosoma* DNA in these taxa should be interpreted cautiously. Finally, species identification and phylogenetic analysis were based on a single genetic marker (ITS1). Although the *T. lewisi*-like genotype showed high nucleotide identity with reference *T. lewisi* sequences and clustered within the *T. lewisi* clade, additional genetic markers would strengthen species-level identification. Moreover, because this genotype was detected in only one animal, its host association and epidemiological significance remain uncertain.

## 5. Conclusions

This multi-year molecular survey provides updated epidemiological information on rodent-associated *Trypanosoma* spp. in wild micromammals in the Republic of Korea. *T. grosi* was the predominant species detected, and, to the best of our knowledge, this study provides the first molecular evidence of a *T. lewisi*-like genotype in a wild micromammal in the ROK. Observed annual variation and the seasonal difference in prevalence should be interpreted cautiously because the sampling design was not fully uniform across the study period. These findings provide baseline information on the occurrence and genetic diversity of rodent-associated *Trypanosoma* spp. in the ROK and highlight the need for further standardized surveillance using broader host sampling, vector investigation, and additional genetic markers.

## Figures and Tables

**Table 1:** Taxonomic composition and *Trypanosoma* spp. positivity among micromammals examined in the Republic of Korea.

Order	Species	No. Examined (%)	No. *T. grosi*- Positive (%)	No. *T. lewisi*-like Genotype- Positive (%)
Rodentia	*Apodemus agrarius*	1010 (86.4)	57 (5.6)	1 (0.1)
	*Apodemus peninsulae*	5 (0.4)	0	0
	*Craseomys regulus*	16 (1.4)	0	0
	*Craseomys rufocanus*	1 (0.1)	0	0
	*Micromys minutus*	24 (2.1)	0	0
	*Microtus fortis*	4 (0.3)	0	0
	*Myodes regulus*	4 (0.3)	0	0
	*Tscherskia triton*	2 (0.2)	0	0
Eulipotyphla	*Crocidura lasiura*	4 (0.3)	0	0
	*Crocidura suaveolens*	2 (0.2)	0	0
	*Crocidura* spp.	96 (8.2)	0	0
	*Mogera robusta*	1 (0.1)	0	0
Total		1169 (100)	57 (4.9)	1 (0.1)

Percentages in the “No. examined” column represent the proportion of the total 1169 micromammals examined, whereas positivity percentages were calculated using the number examined within each taxon as the denominator.

**Table 2:** Prevalence and spatiotemporal distribution of *Trypanosoma* spp. in *Apodemus agrarius* in the Republic of Korea.

Group		No. Examined	No. Positive (%)
Regions	Northern	362	16 (4.4)
	Central	121	8 (6.6)
	Southern	378	22 (5.8)
	Jeju Island	149	12 (8.1)
Year	2022	412	24 (5.8)
	2023	303	4 (1.3)
	2024	91	7 (7.7)
	2025	204	23 (11.3)
Season *	Spring	546	14 (2.6)
	Autumn	373	37 (9.9) **
Total		1010	58 (5.7)

* Seasonal analysis included only 2022, 2023, and 2025, when sampling was conducted during both spring and autumn; samples collected in 2024 were excluded from the seasonal comparison. ** Significantly different from spring based on Fisher’s exact test (*p* < 0.001).

## Data Availability

Data supporting the conclusions of this article are included within the article. The newly generated sequences were submitted to the GenBank database under the accession numbers PZ770430–PZ770449. The datasets used and/or analyzed during the present study are available from the corresponding author upon reasonable request.
